# Pharmacology of Adenosine Receptors: Recent Advancements

**DOI:** 10.3390/biom13091387

**Published:** 2023-09-14

**Authors:** Fabrizio Vincenzi, Silvia Pasquini, Chiara Contri, Martina Cappello, Manuela Nigro, Alessia Travagli, Stefania Merighi, Stefania Gessi, Pier Andrea Borea, Katia Varani

**Affiliations:** 1 Department of Translational Medicine, University of Ferrara, 44121 Ferrara, Italy; chiara.contri@unife.it (C.C.); cppmtn@unife.it (M.C.); manuela.nigro@unife.it (M.N.); alessia.travagli@unife.it (A.T.); mhs@unife.it (S.M.); gss@unife.it (S.G.); vrk@unife.it (K.V.); 2 Department of Chemical, Pharmaceutical and Agricultural Sciences, University of Ferrara, 44121 Ferrara, Italy; psqslv@unife.it; 3 University of Ferrara, 44121 Ferrara, Italy; bpa@unife.it

**Keywords:** adenosine, adenosine receptors, A_1_, A_2A_, A_2B_, A_3_, therapeutic potential

## Abstract

Adenosine receptors (ARs) are widely acknowledged pharmacological targets yet are still underutilized in clinical practice. Their ubiquitous distribution in almost all cells and tissues of the body makes them, on the one hand, excellent candidates for numerous diseases, and on the other hand, intrinsically challenging to exploit selectively and in a site-specific manner. This review endeavors to comprehensively depict the substantial advancements witnessed in recent years concerning the development of drugs that modulate ARs. Through preclinical and clinical research, it has become evident that the modulation of ARs holds promise for the treatment of numerous diseases, including central nervous system disorders, cardiovascular and metabolic conditions, inflammatory and autoimmune diseases, and cancer. The latest studies discussed herein shed light on novel mechanisms through which ARs exert control over pathophysiological states. They also introduce new ligands and innovative strategies for receptor activation, presenting compelling evidence of efficacy along with the implicated signaling pathways. Collectively, these emerging insights underscore a promising trajectory toward harnessing the therapeutic potential of these multifaceted targets.

## 1. Introduction

**Figure 1:**
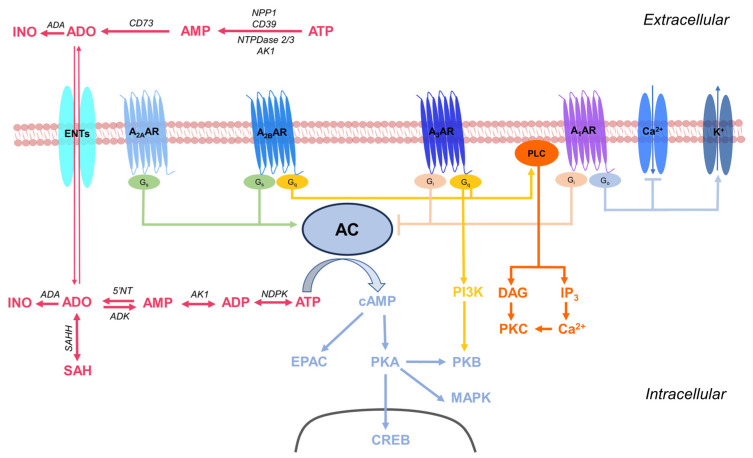
Overview of adenosine metabolism and AR intracellular signaling. Extracellularly, adenosine (ADO) is primarily derived from adenosine monophosphate (AMP) via the catalytic action of ecto-5′-nucleotidase (CD73). Multiple enzymatic pathways, including nucleoside triphosphate diphosphohydrolase-1 (NTPDase1 or CD39), NTPDase2 and NTPDase3, nucleotide pyrophosphatase/phosphodiesterase-1 (NPP1), and adenylate kinase-1 (AK1), contribute to the generation of AMP from ATP. Adenosine transport across the cell membrane is facilitated by equilibrative nucleoside transporters (ENTs). Intracellularly, AMP is converted to adenosine by cytosolic 5′-nucleotidase (5′NT), and the reverse reaction is mediated by adenosine kinase (ADK). Additionally, ATP to ADP and ADP to AMP interconversions are catalyzed by AK-1 and nucleotide diphosphokinase (NDPK), respectively. Adenosine can also be generated from S-adenosylhomocysteine (SAH) through the enzymatic action of SAH hydrolase (SAHH). Its enzymatic degradation occurs via adenosine deaminase (ADA), which converts adenosine to inosine (INO). On the signaling front, A_2A_ and A_2B_ ARs activate adenylyl cyclase (AC) through Gs protein coupling, stimulating the conversion of ATP to cyclic AMP (cAMP). Conversely, A_1_ and A_3_ ARs inhibit AC via Gi protein coupling. The primary downstream effectors of cAMP are cAMP-dependent protein kinase (PKA) and exchange protein directly activated by cAMP (EPAC). PKA primarily regulates transcription through phosphorylation of the cAMP response element-binding protein (CREB). Protein kinase B (PKB) and mitogen-activated protein kinases (MAPK) are common substrates for PKA. Additionally, A_2B_ and A_3_ ARs can activate Gq proteins, thereby stimulating phospholipase C (PLC) and subsequently leading to the formation of diacylglycerol (DAG) and inositol triphosphate (IP3). These molecules activate protein kinase C and elevate intracellular Ca^2+^ levels, respectively. A_3_ARs also regulate the phosphatidylinositol 3-kinase (PI3K)/PKB axis via Gq proteins. A_1_AR activation modulates ion channels, thus inhibiting Ca^2+^ channels and activating K^+^ channels.

Adenosine is a biochemical molecule of paramount importance that performs a diverse range of physiological roles in the organism. It is a nucleoside composed of a purine base, adenine, linked to a ribofuranose moiety through a β-N_9_-glycosidic bond. This molecule is involved in a wide range of biological functions in virtually every system, organ, and tissue of the body. Endogenous adenosine triphosphate (ATP) can be extensively released during processes such as necrosis, apoptosis, inflammation, hypoxia, and mechanical injury. Extracellular adenosine primarily originates from ATP through the actions of the enzymes, nucleoside triphosphate diphosphohydrolase-1 (NTPDase1 or CD39) and ecto-5′-nucleotidase (CD73). However, other enzymatic processes can also play a role in producing extracellular adenosine. These processes encompass the conversion of ATP by nucleotide pyrophosphatase/phosphodiesterase-1 (NPP1), NTPDase2 and 3, adenylate kinase-1 (AK1), followed by the hydrolysis of the resulting adenosine monophosphate (AMP) by CD73 (Figure 1). Inside the cell, the metabolism of adenosine is predominantly regulated by enzymes such as adenosine kinase (ADK) and adenosine deaminase (ADA). In addition, adenosine can also be generated through the hydrolysis of S-adenosylhomocysteine (SAH) by SAH hydrolase (SAHH) [1].

Adenosine, ATP, and other purine and pyrimidine nucleotides signal through purinergic receptors, also known as purinoceptors. These membrane receptors are divided into P1 purinoceptors, which respond to adenosine and are thus generally referred to as adenosine receptors (ARs), and P2 purinoceptors, which respond to ATP and other nucleotides [2]. Currently, four main subtypes of ARs have been identified, namely, A_1_, A_2A_, A_2B_, and A_3_ ARs. A_2A_ and A_2B_ ARs are Gs-protein-coupled receptors, whereas A_1_ and A_3_ ARs are Gi-coupled receptors, although A_2B_ and A_3_ ARs may also interact with Gq proteins (for a comprehensive review of their signal transduction, refer to [3]). These receptors are distributed throughout the body, including the central nervous system (CNS), cardiovascular system, peripheral organs, and immune system. In the central nervous system, adenosine plays an important role in modulating neurotransmission. Activation of ARs in the brain leads to a decrease in neuronal activity, promoting sedation and sleep [4]. Additionally, adenosine has protective effects on the brain by reducing inflammation and preventing neuronal damage. In the cardiovascular system, adenosine contributes to the regulation of blood flow and arterial pressure. Adenosine induces vasodilation of the arteries, thereby increasing blood flow and tissue oxygenation. Furthermore, adenosine also inhibits the contraction of smooth muscle cells in coronary arteries, protecting the heart from ischemia and heart attacks. Adenosine also plays a significant role in regulating the immune response. Activation of ARs on immune cells reduces the production of inflammatory cytokines, thereby decreasing inflammation and the activity of T lymphocytes [5]. This mechanism of immune regulation is crucial for maintaining a balance between an effective immune response and an excessive immune reaction.

**Figure 2:**
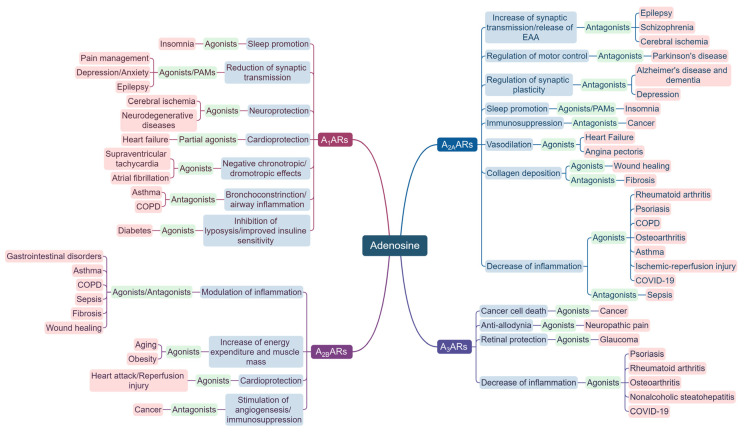
Therapeutic potential of adenosine receptors. The physiological effects of the receptors are in light blue, the pharmacological actions of ligands suitable for treatment are in light green, and the pathologies in which the ligands can be utilized are in pink.

Given its significance in various systems of the body, understanding the mechanisms of adenosine and its receptors unveils new therapeutic perspectives for a broad spectrum of disorders and medical conditions. The purpose of this review is to gather and critically analyze the latest information on the therapeutic potential of adenosine and its receptors (Figure 2). By synthesizing the current knowledge, we aim to provide a comprehensive understanding of the diverse therapeutic possibilities offered by adenosine ligands in various physiological systems and pathological conditions. Additionally, we strive to investigate whether some of the challenges that have hindered the clinical translation of compounds modulating the adenosine system have been, at least partially, resolved.

## 2. ARs in CNS Diseases

Adenosine, derived from ATP catabolism or directly released by neurons and glia, has neuromodulatory actions and regulates numerous physiological functions at the CNS level [6]. The A_1_AR is widely distributed in the CNS and has an inhibitory action on neuronal activities. Activation of A_1_ARs reduces neuronal excitability and can have sedative, analgesic, and anticonvulsant effects. Selective A_1_AR agonists or positive allosteric modulators (PAM) have been studied as potential treatments for epilepsy and other neurological conditions characterized by neuronal hyperactivity [7]. The A_2A_AR is abundant in the basal ganglia and plays an important role in regulating motor tone. Activation of A_2A_ARs can have both neuroprotective and harmful effects, depending on the context [8]. Some compounds acting on A_2A_ARs have been studied as possible pharmacological treatments for neurodegenerative diseases and neuropsychiatric disorders. The A_2B_ARs have been less studied compared to other subtypes, but they are believed to play a role in neuroprotection and the regulation of the neuroinflammatory response. The A_3_ARs are mainly expressed by microglial cells, and their activation can influence the inflammatory response and neuroprotection. 

### 2.1. Recent Advancements in AR Modulation for Pain Management

Neuropathic pain is a widespread and poorly managed problem, resulting from nerve injury and inflammation causing central sensitization and amplified pain. Adenosine and AR ligands effectively reduce neuropathic pain in preclinical models by activating A_1_ARs and/or A_3_ARs, and both A_2A_AR agonists and antagonists have shown efficacy in pain models [4,9]. However, finding a safe and successful way to utilize this pathway for clinical pain treatment remains a challenge. Numerous clinical trials investigating analgesic agents acting as agonists at the A_1_ and A_2A_ ARs were discontinued due to either insufficient efficacy or the occurrence of side effects [10]. The potential CNS and cardiovascular side effects associated with A_1_ or A_2A_ AR agonists could restrict their dose range and overall utility [11].

Recently, several strategies have been implemented to overcome these limitations. These include the development of partial agonists, PAMs, biased agonists, and innovative drug delivery systems. PAMs can augment the responsiveness of ARs to endogenous adenosine specifically within the localized regions of its elevated production [7,12,13]. MIPS521, an A_1_AR PAM, demonstrated in vivo analgesic effectiveness by modulating the elevated endogenous adenosine levels observed in the rat spinal cord during neuropathic pain states. The authors also reported elegant Gaussian accelerated molecular dynamic simulations that offer mechanistic insights into the positive cooperativity of MIPS521. These simulations suggest that the cooperativity is likely achieved by stabilizing the A_1_AR-Gi_2_ complex, which facilitates the formation of, and delays relaxation from, TM6 and TM7 in the ‘G protein-bound-like’ conformation [14]. To further support the role of A_1_ARs in neuropathic pain, in resiniferatoxin-induced neuropathy, Kan et al. found a reduced activity of the transmembrane isoform of prostatic acid phosphatase, which hydrolyzes extracellular AMP into adenosine, and downregulation of A_1_ARs. Low levels of adenosine associated with a low expression of A_1_ARs contributed to the development of mechanical allodynia [15]. Recently, the selective activation of Gαob, one of the six Gαi/o subtypes, by the A_1_AR agonist, benzyloxy-cyclopentyladenosine, was demonstrated to produce analgesic effects in an in vivo model of chronic neuropathic pain without inducing sedation, bradycardia, hypotension, or respiratory depression [16]. This breakthrough represents a significant advancement in creating new research tools and drugs based on the untapped potential of biased and Gα-selective agonists.

Studies using A_2A_ARs agonists delivered to the injured nerve site demonstrated effective suppression of neuropathic pain and neuroinflammation [17]. A single peri-sciatic nerve administration of the A_2A_ARs agonist, ATL313, effectively suppressed ongoing neuropathic pain in rats [18]. This effect appears to be facilitated, at least in part, by the sustained activation of protein kinase A (PKA), the release of the anti-inflammatory cytokine interleukin (IL)-10, the decreased release of IL-1β and nitric oxide, as well as the reduced expression of markers associated with monocytes/macrophages. However, several studies also have indicated that inhibiting A_2A_ARs could alleviate pain in various acute and neuropathic models [19,20,21]. The inconsistencies in the reported effects of A_2A_ARs might arise from the possible conflicting roles that A_2A_ARs play in the periphery versus the CNS.

The A_3_AR has also emerged as a potential therapeutic target for neuropathic pain [22]. Therapeutic intervention with selective A_3_AR agonists could take advantage of the activation of an AR subtype that is less prone to developing side effects compared to A_1_ and A_2A_ ARs. Durante et al. recently provided further insights into the mode of action of A_3_ARs in reducing neuropathic pain. They demonstrated that, while A_3_AR activation led to a reduction of neuropathic pain in wild-type (WT) mice, Rag-knockout (KO) mice lacking T and B cells did not respond to A_3_AR agonist treatment. The anti-allodynic effect of A_3_AR activation was reinstated in Rag-KO mice through the adoptive transfer of CD4^+^ T cells from WT mice. Furthermore, the activation of A_3_AR on CD4^+^ T cells resulted in the release of IL-10, which reduced dorsal root ganglion excitability and regulated neuronal hypersensitivity [23].

### 2.2. Recent Advancements in AR Modulation for Neurodegenerative Diseases

Neurodegenerative diseases represent a group of pathologies characterized by the progressive loss of neurons and cerebral functions, often leading to severe disabilities and cognitive decline. Among these pathologies, Alzheimer’s disease (AD) and Parkinson’s disease (PD), together with autoimmune Multiple Sclerosis (MS), are the most well-known and studied, also in connection with the involvement of ARs.

Preclinical and clinical studies have suggested that modulating ARs could have beneficial effects on neurodegenerative diseases [6,24]. The significant interest in recent years stems from the fact that adenosine is capable of influencing synaptic transmission, neuroinflammation, energy metabolism, sleep–wake cycle, and stress response, all of which are processes altered in neurodegenerative diseases.

#### 2.2.1. Alzheimer’s Disease

Dysregulation of A_2A_ARs appears to play a significant role in neurodegenerative processes, particularly in AD and aging-related cognitive disorders [25]. A_2A_ARs play a role in synaptic remodeling during development and aging, with increased expression in aged individuals and animal models [26,27]. In AD patients and related rodent models, A_2A_AR expression has been found significantly elevated in the cortex [28], hippocampus [29,30], glial cells [31], and platelets [32]. Activation or overexpression of A_2A_ARs has been linked to memory deficits and other aging-like phenotypes [33], while pharmacological blockade or deletion of A_2A_ARs has been shown to mitigate synaptotoxicity and memory deficits induced by β-amyloid (Aβ) peptides in various AD and tauopathy models [34,35,36].

Recent preclinical studies have further corroborated the involvement of A_2A_ARs in AD. Dias and colleagues suggested the role of A_2A_ARs in regulating Ca^2+^ dynamics in astrocytes, where the antagonist, SCH58261, controlled ATP-evoked Ca^2+^ responses, an effect blunted by Aβ1-42 peptides [37]. Aβ1-42-induced synaptic and memory deficits were not encountered in CD73-KO mice, strongly linking cognitive impairment and synaptic dysfunction to ATP-derived adenosine [38].

Playing the role of an AR antagonist, caffeine has been researched regarding its potential as a protective agent against neurodegenerative disorders. Strong epidemiological and experimental evidence substantiates the notion that regular and prolonged caffeine intake can restore synaptic plasticity and alleviate cognitive deterioration in conditions of altered allostatic states, such as AD [39]. A recent and comprehensive work by Paiva et al. performed in mice revealed that chronic caffeine consumption has widespread and diverse effects on the hippocampus, impacting multiple biological processes simultaneously, including epigenomic, proteomic, and metabolomic levels, improving the signal-to-noise ratio during information encoding [40]. In the Tg4-42 mouse model of AD, long-term caffeine consumption resulted in reduced hippocampal neuron loss, improved learning and memory deficits, and enhanced neurogenesis, with no impact on extracellular Aβ levels [41].

#### 2.2.2. Parkinson’s Disease

The approval of istradefylline (also known as KW-6002) as an add-on treatment to levodopa/carbidopa for adult PD patients experiencing “off” episodes signifies a major breakthrough in the application of drugs that interact with the adenosinergic system [42]. Although istradefylline has been approved in the USA and Japan but not in the European Union, this milestone has not only spotlighted the therapeutic potential of the A_2A_AR antagonists but also paved the way for an array of research endeavors and possible therapeutic applications related to this class of compounds [43].

Primarily located in the putamen, caudate, nucleus accumbens, and external globus pallidus, A_2A_ARs interact with dopamine D2 receptors (D2Rs) within the indirect basal ganglia pathway. This explains its potential to modulate motor symptoms in PD. As recently demonstrated, extracellular adenosine, via A_2A_ARs, increases PKA activity in striatal indirect spiny projection neurons and restricts the dopamine-induced rise of PKA activity in striatal direct spiny projection, both actions resulting in reduced locomotion [44]. Thus, adenosine and dopamine appear to form a counterbalancing system, potentially aiding fine motor control.

A recent alternative to istradefylline is KW-6356, a novel A_2A_AR antagonist/inverse agonist. When tested on 1-methyl-4-phenyl-1,2,3,6-tetrahydropyridine (MPTP)-treated common marmosets, KW-6356 demonstrated a remarkable ability to effectively reverse motor disability. Notably, its anti-parkinsonian activity was found to be superior to that of istradefylline, all the while avoiding significant induction of dyskinesia [45]. In the same model, KW-6356 exhibited the ability to augment the anti-Parkinsonian effects of different doses of L-DOPA [46].

Extracellular adenosine is predominantly produced through the catabolism of ATP, facilitated by the ectonucleotidase CD73. This makes CD73 an attractive potential target for pharmacological interventions in PD [47]. By curbing the production of adenosine from CD73, neuroinflammation driven by microglia was significantly reduced, which in turn enhanced the survival of dopaminergic neurons and improved motor function in models of PD. The A_2A_ARs mediated this process, amplifying inflammation by antagonizing the dopamine-mediated anti-inflammatory responses [48]. During the pre-symptomatic phase of a rat model of PD induced by 6-hydroxydopamine, there was an observed increase in ATP release from striatal nerve terminals. This increase led to a rise in adenosine levels through the action of CD73, and subsequently, the activation of A_2A_ARs, influencing corticostriatal long-term potentiation [49]. These findings indicate that the activation of A_2A_ARs plays a crucial role in the abnormal synaptic plasticity associated with the onset of motor symptoms in PD.

Numerous studies have shown that coffee consumption may lower the risk of developing PD [50,51]. It is believed that these beneficial effects are predominantly due to the action of caffeine as an antagonist of the A_2A_ARs. Recently, Ishibashi and colleagues sought to determine the occupancy rates of striatal A_2A_ARs by caffeine, following coffee consumption in individuals with Parkinson’s disease. The study’s conclusion highlighted that a significant A_2A_AR occupancy could be achieved by consuming a cup of coffee, which is approximately equivalent to 100 mg of caffeine [52].

#### 2.2.3. Multiple Sclerosis

A_1_AR activation has been shown to have mostly beneficial effects on MS, by decreasing inflammatory response and promoting remyelination. However, it also increases the permeability of the blood–brain barrier, which makes this treatment approach somewhat uncertain [53]. A_2A_AR is associated with anti-inflammatory effects and can influence the course of MS, with early A_2A_AR activation reducing disease severity [54,55] but late activation worsening it [56,57]. Recently, in the experimental autoimmune encephalomyelitis (EAE) model, Zheng et al. found that A_2A_AR expression increased in the choroid plexus (CP), leading to enhanced CP gateway activity at day 12 post-immunization. Treatment with the A_2A_AR antagonist, KW6002, or focal knock-down of CP-A_2A_ARs reduced T cell trafficking across the CP and alleviated EAE pathology. In cultured CP epithelium, A_2A_AR activation increased the permeability of the CP and facilitated lymphocyte migration [58].

The role of the A_2B_ARs in MS also remains elusive. A recent work by Coppi et al. demonstrated that A_2B_AR activation inhibits oligodendrocyte precursor cell maturation by reducing voltage-dependent K^+^ currents. Silencing A_2B_ARs in cells led to increased cell maturation, decreased sphingosine kinase 1 expression, and enhanced sphingosine-1-phosphate lyase levels [59].

### 2.3. Recent Advancements in AR Modulation for Brain Injury

In the context of brain injury, whether of traumatic, ischemic, or chemical origin, activation of ARs has been shown to exert both neuroprotective and neurotoxic effects, primarily through the regulation of excitotoxicity, inflammation, blood flow, and neuronal survival [60,61].

#### 2.3.1. Traumatic Brain Injury

In traumatic brain injury (TBI), recent works further explored the role of A_2A_ and A_3_ARs. TBI can lead to dysregulated fear memory, contributing to post-traumatic stress disorder and anxiety. In a craniocerebral trauma model, the A_2A_AR agonist, CGS21680, further enhanced fear memory, while the A_2A_AR antagonist, ZM241385, reduced freezing levels. Genetic knockdown of neuronal A_2A_AR in specific hippocampal regions (CA1, CA3, and DG) reduced fear memory after TBI, with the KO in the DG region having the most significant impact [62]. Farr et al. investigated the effect of the selective A_3_AR agonist, MRS5980, on the pathological outcomes and cognitive function in CD1 male mouse models of TBI. MRS5980 reduced secondary tissue injury, brain infarction, and cognitive impairment, specifically linked to reduced activation of the NFκB and the MAPK pathways, as well as the inhibition of the downstream NOD-like receptor pyrin domain-containing 3 (NLRP3) inflammasome activation. Additionally, the use of MRS5980 led to a decrease in the influx of CD4^+^ and CD8^+^ T cells caused by TBI [63].

#### 2.3.2. Cerebral Ischemia

All subtypes of ARs have been studied for their role in cerebral ischemia. A_1_AR agonists and A_2A_AR antagonists exhibit a neuroprotective effect immediately after the insult, while A_2A_ and possibly A_2B_ and A_3_ AR agonists control inflammation and infiltration in the hours and days following brain ischemia, providing protection [64]. As well as in other therapeutic applications, the use of A_1_AR full agonists is hampered by their cardiovascular side effects. Using A_1_AR partial agonists, which are potentially less likely to cause side effects but still have similar effectiveness to full agonists, Martire et al. demonstrated a significant improvement in synaptic transmission in mice subjected to oxygen-glucose deprivation [65]. A recent elegant study aimed to explore the potential of A_1_ARs as imaging biomarkers and treatment targets for stroke using positron emission tomography. After transient middle cerebral artery occlusion, A_1_ARs were found to be overexpressed in microglia and infiltrated leukocytes in the ischemic area. Treatment with the A_1_AR agonist, ENBA, reduced the proliferation of microglia and macrophages. Additionally, A_1_AR activation led to a decrease in brain lesion size, as measured by T2W-MRI, and improved neurological outcomes, including motor, sensory, and reflex responses [66].

The neuroprotective effect of A_2A_AR antagonists was recently corroborated. The targeted inactivation of endothelial A_2A_ARs resulted in a reduction of ischemic brain injury and improvement in post-stroke outcomes. These beneficial effects were achieved, at least partially, by exerting anti-inflammatory effects through the blockade of NLRP3 inflammasome activity, leading to decreased levels of cleaved caspase 1 and IL-1β expression [67]. In rat striatal slices exposed to oxygen and glucose deprivation (OGD), the A_2A_AR antagonist, SCH58261, notably reduced ionic imbalances and the occurrence of anoxic depolarization in medium-spiny neurons. On the other hand, the activation of A_2A_ARs appeared to worsen the damage caused by OGD, potentially by inhibiting K^+^ channels [68]. Although few studies have investigated the role of A_2B_ARs in cerebral ischemia, the findings of a recent work suggested that A_2B_AR activation may represent a new and interesting pharmacological approach. In a rat model of focal ischemia induced by transient middle cerebral artery occlusion, the A_2B_AR agonist, BAY60-6583, improved neurological deficits, significantly reduced brain damage in the cortex and striatum, reduced the activation of microglia and alterations in astrocytes, decreased the expression of TNF-α, increased the expression of IL-10, and reduced the infiltration of blood cells in the ischemic cortex [69]. Regarding A_3_AR, its stimulation with the selective agonist, IB-MECA, improved memory deficits caused by chronic cerebral ischemia in mice, reduced ERK phosphorylation and GFAP expression, and upregulated MAP-2 and IFN-β [70]. In a study using nonhuman primates with transient middle cerebral artery occlusion, the dual A_1_/A_3_ agonist, AST-004, exhibited a reduction in ischemic damage, thereby demonstrating the potential to simultaneously target the two neuroprotective AR subtypes [71].

#### 2.3.3. Chemotherapy-Induced Neurotoxicity

Chemotherapeutic agents often cause neurological impairments. Two recent studies explored the potential neuroprotective role of A_2A_AR blockade or A_3_AR activation in cisplatin-induced neurotoxicities. Oliveros et al. revealed a significant increase in A_2A_AR and related signaling molecules in the adult mouse hippocampus following cisplatin treatment. They observed that blocking the A_2A_ARs with KW-6002 (istradefylline) prevented the negative effects of cisplatin on neural progenitor cell proliferation and dendrite development in newly generated neurons. Additionally, the inhibition of A_2A_ARs improved memory and reduced anxiety-like behavior in the treated mice [72]. A protective effect on cisplatin-induced neurotoxicity was found activating A_3_ARs with the agonist, MRS5980. The compound normalized the expression of different genes that were altered by cisplatin, thereby preventing mitochondrial dysfunction and oxidative stress. Moreover, it upregulated genes associated with repair pathways. MRS5980 also successfully reversed cisplatin-induced cognitive impairment, neuropathy, and sensorimotor deficits [73].

### 2.4. Recent Advancements in AR Modulation for Epilepsy

The fact that adenosine functions as an endogenous anticonvulsant and seizure terminator has been known for decades. The anticonvulsant effects of adenosine are mainly mediated by A_1_ARs due to this receptor’s high affinity for adenosine and its predominant expression in the seizure-prone limbic system [74]. They primarily function by blocking N-type calcium channels and inducing neuronal hyperpolarization. They also suppress neuronal hyperexcitability and maintain an inhibitory tonus in the brain. Increased levels of adenosine and A_1_AR expression were observed following seizures in temporal lobe epilepsy patients, which is believed to be a protective feedback mechanism to limit seizure duration and intensity [75]. A_2A_ARs are known for their excitatory effects, enhancing NMDA receptor function and increasing glutamate release. They tend to have a proconvulsive role, though some studies suggest an anticonvulsive effect. The roles of A_2B_ and A_3_ ARs in epilepsy are not clearly characterized, though it is noted that the activation of A_3_ARs can counteract the inhibitory effects of A_1_ARs [76].

As already stated, targeting A_1_ARs with full agonists is often associated with cardiovascular side effects. Recently, Sagu et al. proposed an intriguing alternative strategy to target A_1_ARs for neurological disorders. A_1_ARs form a complex with the neuronal protein neurabin and the regulator of G protein signaling 4 (RGS4), a protein that inhibits G protein signaling. They developed a peptide that blocks the interaction between A_1_ARs and neurabin, increasing the A_1_AR signaling and consequently reducing kainate-induced seizures and neuronal injury. A protective effect of this peptide was also demonstrated in an AD mouse model of spontaneous seizures, where it reduced epileptic spike frequency [77]. A distinct approach has been employed involving deep brain stimulation (DBS), which has proven to be an effective therapy for patients with epilepsy resistant to conventional drugs. It has been demonstrated that the A_1_AR antagonist, DPCPX, reversed the impact of DBS on interictal epileptic discharges in a model of status epilepticus induced by pilocarpine. Furthermore, DBS inhibited the overexpression of ADK, a crucial negative regulator of adenosine, and the downregulation of A_1_ARs [78].

A role for A_2A_ARs in sudden unexpected death in epilepsy (SUDEP) has been recently suggested. In a boosted-KA model of SUDEP using genetically modified ADK knockdown mice, the A_2A_AR antagonist, SCH58261, increased theta and beta oscillations. Additionally, it partially restored the KA injection-induced suppression of gamma oscillation in the nucleus tractus solitarius of epileptic WT mice [79].

### 2.5. Recent Advancements in AR Modulation for Neuropsychiatric Disorders

In the context of neuropsychiatric diseases, the latest studies have primarily focused on the A_2A_ARs. This is because the A_2A_AR plays a significant role in regulating the function of essential neurotransmitters, the dysregulation of which is commonly observed in various neuropsychiatric disorders. A_2A_ARs form functional complexes with dopamine D2Rs and are involved in controlling glutamate and GABA release. Furthermore, A_2A_ARs also play a role in neuroinflammation, which is increasingly recognized as a critical factor in many neuropsychiatric disorders [80,81].

#### 2.5.1. Depression

A subpopulation of lateral septum GABAergic neurons expressing A_2A_ARs was identified as mediating depressive symptoms through direct projections to the lateral habenula and dorsomedial hypothalamus. Additionally, A_2A_AR expression was found to be upregulated in the lateral septum in two male mouse models of repeated stress-induced depression, suggesting that A_2A_AR antagonists could have antidepressant potential [82]. A_2A_AR expression was also found to be upregulated in rats with sevoflurane-induced depression. Activation of A_2A_ARs led to decreased ERK phosphorylation, reduced synaptic plasticity, and the induction of depressive-like behavior [83].

#### 2.5.2. Anxiety

Two recent studies have investigated the effect of chronic or acute caffeine intake on anxiety. Mice exposed to chronic caffeine intake in their drinking water exhibited heightened anxiety-like behavior and improved memory function. Memory enhancement caused by caffeine was prevented when dorsal hippocampal A_2A_ARs were disrupted, while the impact of caffeine on anxiety was blocked when ventral hippocampal A_2A_ARs were deleted. Optogenetic activation of dorsal or ventral hippocampal A_2A_ARs reversed the behavioral changes induced by caffeine [84]. Rats with high anxiety-like behavior, following acute caffeine intake, displayed reduced risk-taking in the multivariate concentric square field test, along with increased BDNF expression in the hippocampus and lower A_2A_AR mRNA expression in the caudate putamen [85].

#### 2.5.3. Schizophrenia

The adenosine hypothesis of schizophrenia suggests that the hyperdopaminergic state typically associated with the condition could be caused by either reduced levels of adenosine in the brain or changes in the density and functional interaction of A_2A_ARs with D2Rs [86,87]. A recent study provides evidence supporting the latter mechanism, using the phencyclidine (PCP) mouse model and A_2A_AR-KO mice. A_2A_AR-KO mice exhibited reduced prepulse inhibition, a characteristic sensory gating impairment seen in schizophrenia, and upregulation of striatal D2Rs without changes in A_2A_AR expression in PCP-treated animals. Furthermore, PCP-treated animals showed a significant reduction in striatal A_2A_AR-D2R heteromers, an effect counteracted by sub-chronic doses of antipsychotic drugs haloperidol or clozapine. Finally, in the caudate nucleus of postmortem brain samples from individuals with schizophrenia, a substantial reduction in A_2A_AR-D2R heteromers was observed [88]. In a subsequent study, the same authors found an increase in A_2A_AR-D2R heteromerization following the exposure of mammalian cells to haloperidol or aripiprazole, and a reduction with clozapine. Using computational binding models, distinctive molecular signatures for each drug were highlighted, explaining their differing effects on heteromerization [89]. In rats treated with methylphenidate to induce mania-like behavior, the A_2A_AR antagonist, SCH58261, reduced locomotor hyperactivity, risk-taking behavior, dopamine, and glutamate levels. It also suppressed PKC-α expression and modulated Akt/GSK-3β/β-catenin axis, indicating the potential of A_2A_AR as a therapeutic target for mania-like behavior treatment [90].

### 2.6. Recent Advancements in AR Modulation for Sleep Disorders

Adenosine plays a critical role in the homeostatic regulation of sleep and wakefulness. A_1_ARs promote sleep by inhibiting wake-promoting neurons and disinhibiting sleep-active neurons, while also mediating homeostatic sleep pressure through astrocytic gliotransmission. A_2A_ARs promote sleep by inhibiting the major arousal systems in the brain, and their inhibition is the main reason for the wake-promoting effects of caffeine [91].

Recently, some noteworthy studies have provided further clarity on the role and involvement of adenosine in sleep. Using a genetically encoded GPCR-activation-based sensor for adenosine, Peng et al. discovered that, in the mouse basal forebrain, the extracellular adenosine concentration was higher during wakefulness compared to non-rapid eye movement (NREM) sleep. A significant increase in adenosine levels was observed during REM sleep, and adenosine concentrations changed rapidly during transitions between different brain states, indicating a release dependent on neural activity. The authors found a correlation between the activation of glutamatergic neurons and changes in extracellular adenosine concentration. When these neurons were ablated, there was a reduced increase in extracellular adenosine during wakefulness and REM sleep, leading to increased wakefulness and impaired sleep homeostasis [92]. In an elegant work, Jagannath and colleagues reveal a regulatory mechanism involving adenosine that enables the coordination of sleep and circadian processes to optimize sleep/wake timing in mice. In particular, adenosine influenced the circadian clock through A_1_/A_2A_ AR signaling, which activates pathways that play a crucial role in regulating the clock genes *Per1* and *Per2* [93].

To further support the sleep-promoting effect of A_1_ARs, their correlation with the hypnotic effect of rosmarinic acid was investigated. In mice, rosmarinic acid decreased neuronal activity in wake-promoting brain regions and increased activity in the sleep-promoting region, resulting in reduced sleep fragmentation and decreased time to enter NREM sleep. These effects were demonstrated to be mediated by its agonistic binding to A_1_ARs [94].

To harness the potential of A_2A_ARs, a PAM was explored as a means to improve insomnia while minimizing cardiovascular side effects often seen with direct agonists. The compound, named A_2A_RPAM-1, was found to effectively alleviate insomnia linked to mania- or schizophrenia-like behaviors in mice. Unlike diazepam, it did not lead to abnormal sleep patterns [95]. Furthermore, activating A_2A_ARs in the olfactory tubercle promoted NREM sleep [96]. A recent study proposed a new understanding of sleep regulation involving astrocytes within the ventrolateral preoptic nucleus. According to this research, astrocytes release ATP in this brain region, which is then converted into adenosine by tissue-nonspecific alkaline phosphatase. Adenosine subsequently inhibits local GABAergic neurons, resulting in augmented excitability GABAergic projection neurons that facilitate sleep [97].

### 2.7. Recent Advancements in AR Modulation for Eye Diseases

In the mammalian eye, ATP and adenosine are crucial for vascular remodeling, retinal function, and neurovascular coupling. A_1_ and A_3_ ARs are generally considered to have protective effects on the retina, while A_2A_ARs modulate neuroinflammation. Due to these roles, there have been developments of A_1_ and A_3_ AR agonists, as well as A_2A_AR agonists or antagonists, as potential treatments for eye diseases such as glaucoma, diabetic retinopathy, and age-related macular degeneration [98].

In a model of glaucoma, the intravitreal injection of the selective A_3_AR agonist, 2-Cl-IB-MECA, reversed the alterations induced by ocular hypertension, preserved retinal ganglion cell (RGC) function, improved retrograde axonal transport, and enhanced optic nerve structure [99]. In a follow-up study, a biodegradable intraocular implant with a porous structure, loaded with 2-Cl-IB-MECA, was developed. The primary goal was to avoid multiple intravitreous injections. The A_3_AR agonist, when released from the implant, effectively maintained its efficacy in reducing retinal cell death and promoting the survival of RGCs induced by transient ischemia [100]. Supporting the therapeutic potential of A_2A_AR antagonists, a recent study investigated the effects of KW6002 on retinal injury induced by the mitochondrial oxidative phosphorylation uncoupler CCCP. KW6002 treatment partially reversed CCCP-induced reduction in retinal thickness and retinal ganglia cell number by increasing mitochondrial content and reducing apoptosis of retinal ganglia cells. Additionally, KW6002 reversed the alterations in the competing endogenous RNA network caused by CCCP treatment [101]. In Thy1-YFPH transgenic mice, A_2A_ARs were studied for their role in regulating the morphogenesis of three types of RGCs during postnatal development and neonatal inflammation. KW6002 had bidirectional effects on dendritic complexity of Type I and III RGCs and altered their morphologies. Under neonatal inflammation, KW6002 increased the proportion of Type I and II RGCs with specific changes in their morphology [102]. Table 1 shows the main recent studies investigating the effects of AR ligands in in vivo models of CNS pathology.

## 3. ARs in Cardiovascular and Metabolic Diseases

Adenosine plays a multifaceted role in cardiovascular function, acting as a regulator that finely adjusts various processes. It helps maintain a balanced cellular energy state and enhances the cells’ ability to withstand stress and injury. Through the interactions with all its receptor subtypes, adenosine influences every key aspect of cardiovascular function. This includes regulating the heart rate and contractility, controlling the conduction of electrical impulses within the heart, modulating autonomic control of the heart, ensuring adequate coronary blood flow, participating in cardiovascular growth and remodeling processes, and providing protection to the heart and blood vessels against harmful insults [103]. Moreover, ARs can influence glucose and lipid homeostasis. Therefore, agonists and antagonists of ARs have been explored as potential treatments for atherosclerosis, diabetes, obesity, and non-alcoholic fatty liver disease in preclinical and clinical studies [104,105]. 

### 3.1. Recent Advancements in AR Modulation for Ischemic Heart Disease

Adenosine has been extensively studied as a mediator of cardiac protection during ischemia-reperfusion [106]. Adenosine is also involved in adaptive preconditioning responses that induce prolonged shifts in stress resistance and limit later remodeling changes and heart failure progression. A recent study investigated the effects and the mechanisms associated with remote tissue compression in a mouse model of myocardial infarction. It was found that rhythmic compression on the forelimb, known as remote conditioning, served as a novel cardioprotective intervention. The study unveiled that the transmission of cardioprotective signals from the compressed limb to the heart relied on the release of adenosine, which acted on A_2A_ and A_2B_ ARs and modulated the cAMP/PKA/NF-κB axis [107]. In a unique porcine model of circulatory arrest and extracorporeal cardiopulmonary resuscitation, it has been shown that the use of the specific A_2A_AR agonist, ATL1223, significantly reduced the severity of systemic ischemia-reperfusion injury, and ameliorated renal, hepatic, and cardiac injury [108]. A different strategy to harness the cardioprotective effects of adenosine against ischemia and reperfusion injury involves prolonging the presence of its elevated concentrations in the extracellular space. The termination of extracellular adenosine signaling occurs when it is taken up into cells by equilibrative nucleoside transporters (ENTs). In a study by Ruan et al., mice exposed to myocardial ischemia and reperfusion injury were treated with the nonspecific ENT inhibitor dipyridamole, resulting in a reduction of myocardial injury. The specific deletion of A_2B_AR in myeloid cells and the myocyte-specific deletion of ENT1 revealed an unanticipated role for myocyte-specific ENT1 in cardioprotection, enhancing myeloid-dependent A_2B_AR signaling during reperfusion [109].

### 3.2. Recent Advancements in AR Modulation for Hypertension

ARs play a role in blood pressure regulation. While A_1_ARs cause vasoconstriction in the renal microcirculation, aorta, and mesenteric arteries, while also stimulating sodium reabsorption in renal tubules, A_2A_ARs induce vasodilation in various vascular beds. The activation of the A_2B_ARs can have complex effects on blood pressure, with both vasodilatory and vasoconstrictive actions depending on specific conditions and physiological context [110].

A_1_AR-induced vascular contractions in mesenteric arteries and aorta were enhanced in L-NAME hypertensive mice, where a higher receptor expression was identified. Cyp4A appeared to play a role in the altered vascular responses of A_1_ARs in mesenteric arteries [111].

The effects of salt diets on blood pressure in salt-sensitive hypertension with A_1_, A_2A_, or A_2B_ AR-KOs were recently investigated. A_2A_AR-KOs showed higher blood pressure, while A_1_AR-KOs and A_2B_AR-KOs had lower blood pressure on the 4% salt diet. While both sexes of A_2A_AR-KOs were more salt-sensitive, female A_1_AR-KOs and A_2B_AR-KOs were less susceptible to salt-induced stroke and had improved survival [112].

A_2A_AR plays a crucial role in the function of brown adipose tissue (BAT) and pathological cardiac remodeling. An endocrine role of BAT in hypertensive cardiac remodeling through the A_2A_AR/fibroblast growth factor 21 (FGF21) pathway has been proposed. It was found that dysfunctional interscapular BAT caused by A_2A_AR-KO leads to accelerated cardiac remodeling in hypertension compared to WT mice. The FGF21, induced by the AMPK/PGC1α pathway in brown adipocytes, is necessary for A_2A_AR-mediated inhibition of hypertensive cardiac remodeling. Administration of recombinant FGF21 improves cardiac remodeling in hypertensive mice lacking interscapular BAT. Additionally, specific A_2A_AR-KO in brown adipocytes inhibits FGF21 production and accelerates cardiac damage in hypertension [113].

### 3.3. Recent Advancements in AR Modulation for the Regulation of Angiogenesis

The role of A_2A_AR activation as a potent angiogenic stimulus is widely recognized [114]. However, the role of the other ARs remains less clear.

A novel anti-angiogenic mechanism based on adenosine production acting on A_2B_ARs has been proposed. Mesenchymal stem cells stimulated with pro-inflammatory cytokines secreted anti-angiogenic extracellular vesicles (EVs) that were enriched in CD73. These EVs inhibited endothelial cell migration and reduced vascularization in in vivo models. The anti-migratory effect of EVs is attributed to oxidative stress induced by NADPH oxidase 2 activation, triggered by adenosine produced by EVs through CD73 activity, via the activation of A_2B_ARs [115].

In fetal intrauterine growth restriction (IUGR) placenta, where abnormal angiogenesis is significant, a recent report revealed reduced adenosine concentration and downregulated expression of A_2A_ARs. Furthermore, the activation of A_2A_ARs in IUGR mice placenta promoted angiogenin-dependent angiogenesis through the phosphorylation of STAT3 and Akt [116]. The administration of adenosine in the diet of piglets with IUGR resulted in an increase in average birth weight and placental efficiency and promoted angiogenesis [117].

### 3.4. Recent Advancements in AR Modulation for Metabolic Diseases

Fascinating new discoveries regarding the enigmatic A_2B_AR have been revealed in a study that examined its potential in combating obesity and aging. Mice with specific deletion of A_2B_ARs in skeletal muscle showed signs of sarcopenia, decreased muscle strength, and reduced energy expenditure (EE), whereas deletion of A_2B_ARs in adipose tissue worsened age-related effects and decreased EE in BAT. Pharmacological activation of A_2B_ARs mitigated obesity induced by a high-fat diet by positively influencing whole-body EE. Additionally, A_2B_AR treatment led to increased muscle mass and force, enhanced the thermogenic capacity of BAT, and promoted browning of white adipose tissue (WAT). A_2B_AR expression was associated with increased EE in human BAT and browning of WAT, suggesting that individuals with low A_2B_AR levels might have been more susceptible to obesity. In human myocytes, A_2B_AR activation improved muscle quality, including fiber composition, oxidative metabolism, glucose uptake, and energy utilization [118]. A_2A_AR has also recently been associated with adipose browning. Kong and colleagues identified complement C3a receptor and C5a receptor as important regulators of adipocyte browning and energy balance. The loss of these receptors promotes the accumulation of regulatory T cells (Tregs), which in turn produce adenosine, which is then converted to inosine. Inosine activates A_2A_ARs, promoting adipocyte browning and attenuating diet-induced obesity [119].

A_1_ARs are widely expressed in adipose tissue and play a significant role in glucose homeostasis. In preclinical studies, A_1_AR agonists reduced lipolysis, improved insulin resistance in rats fed a high-fat diet, lowered plasma triglycerides and cholesterol levels, and enhanced glucose uptake in skeletal muscles. The A_1_AR antagonist, BW-1433, improved glucose tolerance and increased lipolysis [104]. Recent research revealed that insulin resistance induced by a high-sucrose diet is linked to higher levels of A_1_, A_2A_, and A_2B_ ARs in the skeletal muscle, an increase in A_1_ARs in the liver and adipose tissue, and a decrease in A_2B_ARs in the liver. When A_1_ARs were blocked in adipose tissue, insulin signaling improved in control animals, but it had a negative effect on insulin signaling in animals on the high-sucrose diet. A_2A_ or A_2B_ ARs antagonists were found to reverse the impaired insulin signaling in the skeletal muscle of rats on the high-sucrose diet. However, they did not have any significant impact on insulin signaling in the liver or adipose tissue [120]. Table 2 summarizes the most recent in vivo studies using AR ligands concerning cardiovascular and metabolic diseases.

## 4. ARs in Inflammation and Autoimmunity

Adenosine exerts significant control over the inflammatory process. The immunoregulatory effects of adenosine and its receptors, primarily anti-inflammatory in nature, contribute to an overall tissue-protective action [5]. All immune cells of the innate system express the four subtypes of ARs. When A_2A_, A_2B_, and A_3_ ARs are activated in macrophages, they limit the production of various pro-inflammatory mediators while promoting the release of anti-inflammatory ones. Adenosine also plays a regulatory role in dendritic cells (DCs), with A_2A_ARs reducing pro-inflammatory cytokines. In mast cells, A_2B_ARs trigger degranulation, while A_3_ARs display anti-inflammatory properties. Neutrophils express all four ARs: activation of A_1_ and A_3_ ARs promotes chemotaxis and phagocytosis, while A_2A_ and A_2B_ ARs inhibit neutrophil trafficking and effector functions. Adenosine, produced by regulatory Tregs, reduces NF-κB activation in T effector cells through stimulation of A_2A_ARs. Moreover, adenosine modulates B cell functions, with all four receptor subtypes being expressed [121]. Platelets express only A_2A_ and A_2B_ ARs, and when A_2A_AR are activated, they inhibit the secretion of pro-inflammatory mediators, reduce cell activation, and decrease P-selectin expression [122]. New research highlights and corroborates the importance of AR modulation in the regulation of inflammation and in autoimmunity diseases.

### 4.1. Recent Advancements in AR Modulation for Autoimmunity Diseases

The adenosine-based targeting of certain widely used drugs for treating rheumatic diseases, particularly methotrexate, is well recognized [123]. Human synoviocytes exhibit significant expression of both A_2A_ and A_3_ ARs, and their activation induces an anti-inflammatory response [124]. Furthermore, A_2A_ and A3 ARs have been found overexpressed in immune cells from rheumatoid arthritis [125,126], systemic lupus erythematosus [127], and ankylosing spondylitis [128].

A_2A_ or A_2B_ AR stimulation has shown a beneficial effect in various preclinical models of arthritis [126,129,130,131]. Recently, it has been shown that the A_2A_AR agonist, CGS21680, inhibited arthritis development and redirected the differentiation of autoreactive CD4^+^ T cells away from the germinal center T follicular helper lineage. In addition, CGS21680 treatment prevented the emergence of high-affinity glucose-6-phosphate isomerase-specific and IgG1 isotype class-switched polyclonal plasmablasts, resulting in decreased levels of anti-GPI IgG1 antibodies [132]. A distinct response to A_2A_AR stimulation with CGS21680 has been observed in macrophages: it reduced matrix metalloproteinase (MMP) 8 expression in healthy macrophages, but it was unable to decrease MMP8 expression in macrophages from patients with ankylosing spondylitis [133].

An elevated expression of A_2A_AR has been identified in CD11c^+^T-bet^+^ B cells [134], a specific type of B cell that plays a critical role in autoimmunity, particularly in the development of systemic lupus erythematosus. In lupus-prone mice, CGS21680 treatment depleted CD11c^+^T-bet^+^ B cells, CD138^+^ B cells, and pathogenic lymphocytes and reduced anti-nuclear antibodies. Additionally, it decreased kidney pathology and lymphadenopathy and improved the overall condition of the animals, even after the disease onset [135].

In an effort to minimize potential adverse reactions resulting from the widespread distribution of ARs, recent studies focused on creating and assessing a skin-targeted method for delivering an A_3_AR agonist. This agonist is activated by blue light, facilitated by a photo-cleavable masking group. The A_3_AR agonist, known as MRS7344, effectively hindered the development of psoriatic-like characteristics in an IL-23 animal model. This successful outcome illustrates the practicality of utilizing light-directed approaches for treating psoriasis [136]. In a Phase II clinical study conducted on patients with psoriasis, the A_3_AR agonist, piclidenoson (also known as CF101, IB-MECA), was found to be safe and showed effectiveness, leading to significant improvements in skin lesions [137].

Deficiency of adenosine deaminase 2 (DADA2) is caused by ADA2 gene mutations, resulting in an autoinflammatory systemic vasculitis, where neutrophil extracellular traps (NETs) significantly contribute to the disease. Adenosine, through the engagement of A_1_ and A_3_ ARs, was found to trigger NET formation, particularly in neutrophils from female DADA2 patients. In contrast, A_2A_ARs activation had an opposite effect, reducing NET formation, as well as inhibiting cytokine release mediated by NF-κB activation in macrophages derived from DADA2 patients [138].

### 4.2. Recent Advancements in AR Modulation for Osteoarthritis

Evidence suggests that adenosine is crucial for maintaining cartilage structure and function. Its primary role in cartilage homeostasis is acting as a buffer against the inflammatory environment on chondrocytes. Numerous studies have focused on the A_2A_AR, identifying it as the main mediator of adenosine’s protective effects [139]. A_2A_AR or ecto-5′nucleotidase KO mice develop spontaneous OA, and deleting A_2A_AR from chondrocytes has determined an osteoarthritis (OA) phenotype with increased MMP13 and Col10a1 expression. Additionally, injecting adenosine-containing liposomal suspensions intra-articularly prevents OA development in rats [140]. In a subsequent and recent study, A_2A_AR stimulation reduced senescence markers in chondrocytes in vitro and in obesity-induced OA mice. A_2A_AR agonism enhanced the Sirt1/AMPK pathway and increased the anti-senescent p53 variant, Δ133p53α [141]. Furthermore, primary murine chondrocytes from A_2A_AR^−/−^ null mice, which develop spontaneous OA, have mitochondrial dysfunctions. Treatment with the A_2A_AR agonist, CGS21680, improved mitochondrial stability and function in IL-1β-exposed chondrocytes and in an obesity-induced OA mouse model [142].

Forkhead box O (FoxO) transcription factors, stress-responsive mediators, are crucial for maintaining articular cartilage homeostasis. Evidence from mouse FoxO KOs shows that their absence leads to early OA and reduced cartilage autophagy. A_2A_AR stimulation with the agonist, CGS21680, activated FoxO1 and FoxO3, promoting increased autophagy and improved metabolic function in chondrocytes. Enhanced activation of FoxO1 and FoxO3, along with increased autophagic flux, was demonstrated in vivo after administering the liposome-associated A_2A_AR agonist in an obesity-induced OA mouse model [143].

A potential role for A_3_AR in OA was recently suggested. Orally administered A_3_AR agonist, CF101, in OA rats induced by anterior cruciate ligament transection surgery reduced OA cartilage damage, pain, and cartilage pyroptosis. Mechanistically, CF101 inhibited ROS production, NLRP3 inflammasome activation, and gasdermin D cleavage in rat primary chondrocytes, indicating the inhibition of pyroptosis [144].

### 4.3. Recent Advancements in AR Modulation for Respiratory Diseases

The role of adenosine signaling is pivotal in the lung’s response to injuries. Initially, adenosine plays a beneficial anti-inflammatory and tissue-protective role, mainly by activating the A_2A_ and A_2B_ ARs during acute lung injury [145]. However, in chronic respiratory diseases, it triggers the activation of A_1_, A_2B_AR, and A_3_ ARs, leading to a pro-inflammatory state and uncontrolled tissue remodeling [146]. One of the extensively researched A_2B_AR antagonists in asthma and chronic obstructive pulmonary disease (COPD) is CVT-3883. It has been demonstrated to be as efficacious as montelukast, as it effectively lowered the count of inflammatory cells in bronchoalveolar lavage fluid and inhibited the production of proinflammatory mediators originating from macrophages [147]. Novel and potent A_2B_AR antagonists continued to be developed, demonstrating efficacy in an in vivo model of allergic asthma [148]. Nevertheless, the results are still controversial, and more research is required.

In a recent study, the oral A_1_AR antagonist, PBF-680, abrogated the late asthmatic response and reduced the early allergic response, fractional exhaled nitric oxide, and blood eosinophils in mild-to-moderate atopic asthmatics [149]. In asthmatic patients, it also reduced AMP airway hyperresponsiveness [150].

Xiao et al. identified adenosine as a key regulator that suppresses the responses of group 2 Innate Lymphoid Cells (ILC2s) and alleviates allergic airway inflammation. After exposure to the protease papain, levels of adenosine in the lungs were found to be elevated, and the A_2A_ARs were abundantly expressed in lung ILC2s. The AR agonist, NECA, significantly subdued ILC2 responses and mitigated inflammation caused by IL-33 or papain. However, in A_2A_AR-KO mice or when adenosine synthesis was blocked, the inflammation worsened [151]. The DNA-derived drug, Polydeoxyribonucleotide (PDRN), which acts as an A_2A_AR agonist, has recently been the subject of much research. In a rat acute lung injury model induced by lipopolysaccharide (LPS), PDRN effectively reduced lung tissue damage, pro-inflammatory cytokines, apoptotic factors, and MAPK/NF-κB activation [152].

The anti-inflammatory effect of A_2A_AR activation has been recently hypothesized as a strategy to reduce lung inflammation in coronavirus disease 2019 (COVID-19) patients [153,154]. Patients with mild and severe cases of COVID-19 exhibited reduced extracellular adenosine levels, which were linked to elevated concentrations of pro-inflammatory cytokines and were attributed to altered expression of CD39 and CD73 in the T cells of COVID-19 patients [155]. It has been also proposed that the exacerbation of inflammation by oxygenation may result from the absence of hypoxia-induced A_2A_AR activation. Therefore, a direct approach might involve pairing oxygen ventilation for COVID-19 patients with the administration of inhaled adenosine or A_2A_AR agonists [156]. In a study involving 14 COVID-19 patients who received inhaled adenosine, 13 exhibited positive outcomes and a reduction in respiratory symptoms [157].

A link between sevoflurane and A_2B_ARs for the reduction of acute pulmonary inflammation has been described. Sevoflurane reduced LPS-induced polymorphonuclear neutrophil (PMN) infiltration and edema in WT mice. Chimeric mice expressing A_2B_ARs exclusively on leukocytes showed decreased PMN counts after sevoflurane treatment [158]. Recent research demonstrated that A_2B_AR activation by adenosine is the mechanism by which cupping, a traditional Chinese alternative therapy, attenuates LPS-induced lung inflammation [159].

A selective A_3_AR and partial PPARγ agonist, LJ-529, significantly improved pulmonary emphysema induce by elastase, restoring pulmonary function, reducing airspace enlargement, MMP activity, and apoptosis [160]. Activation of A_3_ARs was recently proven beneficial in a bleomycin murine model of lung fibrosis. The A_3_AR selective agonist, MRS5980, attenuated bleomycin-induced lung stiffness, TGF-β levels, α-SMA deposition, and inflammatory and oxidative stress markers [161].

In a model of bronchopulmonary dysplasia induced by hyperoxia exposure, caffeine treatment reduced lung injury and enhanced alveolar development by decreasing oxidative stress and inflammatory infiltration. Mechanistically, caffeine reduced NLRP3 activity, NF-κB pathway activation, and also downregulated the expression of A_2A_AR protein in the lungs of mice [162].

### 4.4. Recent Advancements in AR Modulation for Sepsis

The anti-inflammatory and immunosuppressive effect of adenosine is evidently counterproductive in sepsis. During systemic inflammation or tissue damage, extracellular adenosine levels increase significantly. Septic shock patients exhibit a tenfold rise in plasma adenosine concentrations due to reduced ADA and ADK activity and increased CD73 activity. The immunosuppressive effects of adenosine are mainly mediated by A_2A_AR, but also by A_2B_ARs, whose expression is rapidly increased by endotoxin or inflammatory mediators. On the other hand, stimulating A_1_ or A_3_ ARs during sepsis may have advantageous effects, leading to a reduction in mortality and mitigating renal and hepatic damage [163].

A recent significant publication delineated the function of adenosine and CD39^hi^ plasmablasts as crucial factors in the induction of immunosuppression caused by sepsis. The study revealed that sepsis led to an enlargement of a B cell group expressing CD39, resulting in an increase in immunosuppressive adenosine. This adenosine, in turn, interacted with A_2A_ARs, weakening the bactericidal action of macrophages and boosting the production of IL-10 [164]. In the same model of sepsis induced by cecal ligation and puncture, the A_2A_AR antagonist, ZM241385, enhanced survival by improving bacterial clearance. However, when Treg-deleted mice were treated with ZM241385, there was no improvement in sepsis survival, indicating that the effect relies on Treg activity. Inactivating A_2A_AR led to decreased frequencies and impaired function of Foxp3^+^ Tregs.

Novel insights into the role of A_2B_ antagonism in sepsis were also gained. Inhibition of chemokine receptors CXCR4 and CXCR7 decreased platelet–neutrophil complex formation in WT mice, but such protective anti-inflammatory effects were not observed in A_2B_AR^−/−^ animals [165]. In mouse models of zymosan- and fecal-induced peritonitis, sevoflurane showed protective effects in WT animals but not in mice lacking A_2B_ARs. The presence of A_2B_AR expression on both hematopoietic and nonhematopoietic compartments was necessary for sevoflurane’s protective effects [166]. Table 3 shows the most recent studies evaluating the effects of AR ligands in in vivo models of inflammatory and autoimmune diseases.

## 5. ARs in Cancer

Immunotherapy has revolutionized cancer treatment by harnessing the immune system’s ability to target and eliminate cancer cells in a specific manner. The tumor microenvironment (TME) can experience transient or chronic intratumoral hypoxia, leading to metabolic changes and the CD39/CD73-mediated accumulation of adenosine derived from ATP. This accumulation promotes A_2A_/A_2B_ AR-dependent suppression of the host’s immune defense mechanisms, including the recruitment and differentiation of Treg cells and the inhibition of effector immune cells such as T cells, NK cells, macrophages, and DCs [167]. Because of this, adenosine and its receptors represent one of the main mechanisms through which tumor cells evade immune surveillance [168]. Furthermore, adenosine does not only affect immune cell responses to cancer cells but also influences tumor angiogenesis, lymphangiogenesis, cancer-associated thrombosis, and tumor perfusion. With the intention of exploiting these mechanisms, in recent years, there has been a surge in preclinical and clinical studies delving into the potential of the adenosinergic system for cancer immunotherapy, undoubtedly making it one of the most rapidly advancing frontiers in adenosine pharmacology [169]. Many clinical trials have explored the use of monoclonal antibodies or small molecule inhibitors that target the CD39/CD73/A_2A_AR pathway either as standalone treatments or in combination with anti-PD-1/PD-L1 therapies [170]. Preclinical data, however, indicate a potential role for other receptor subtypes as well.

### 5.1. Recent Advancements in A_2A_AR Modulation for Cancer

Recent experimental evidence strongly corroborates the role of A_2A_AR inhibition in restoring T cell effector function. Using a single-cell reporter strategy, it was found that A_2A_AR antagonism enhances cytotoxic T cell contact stability, improves lytic granule polarization and exocytosis, and increases the delivery of sublethal perforin hits per cytotoxic T cell contact. Moreover, A_2A_AR antagonism restored the functionality of tumor-infiltrating cytotoxic T cells in a melanoma model, leading to a local effector phenotype characterized by prolonged dwell time and improved sublethal hit delivery [171]. Research into the signaling pathway through which adenosine undermines the immune competence of peripheral T cells and lymphocytes infiltrating tumors has revealed that A_2A_AR activation, through PKA activation, disrupts the TCR/mTORC1 signaling in human CD8^+^ T cells. This impairment subsequently hampers both metabolic efficiency and effector functionalities [172]. In a model of chronic lymphocytic leukemia, the use of the A_2A_AR antagonist, SCH58261, demonstrated the restoration of immune competence. This was achieved by inhibiting the accumulation and differentiation of Treg cells, reinstating effective T cell functions, and shifting monocytes toward an inflammatory (M1-like) phenotype [173]. Monocytes/macrophages constitute a vital component within tumor tissues, playing a pivotal role in tumor progression and therapeutic response. Within human hepatocellular carcinoma (HCC) tissue, macrophages exhibited heightened proliferative potential induced by adenosine derived from the tumor itself. These rapidly dividing macrophages show reduced differentiation, display immunosuppressive characteristics, and their presence is inversely linked to the prognosis of HCC patients. The study revealed that autocrine granulocyte-macrophage colony-stimulating factor upregulated A_2A_AR expression in macrophages, working in synergy with adenosine to stimulate their proliferation [174]. Research has demonstrated that adenosine hampers the maturation process and hinders the antigen presentation function of CD103^+^ DCs, a crucial stage in promoting anti-tumor immune responses. The suppressive characteristics triggered by adenosine in human DCs, which lead to immune tolerance, were effectively counteracted by using the A_2A_AR antagonist, AZD4635 [175]. Using a syngeneic B cell lymphoma model, conditional deletion of A_2A_ARs in myeloid cells augmented the therapeutic efficacy of anti-CD20 mAb [176].

The detrimental role of A_2A_AR activation in cancer does not end with its effects on immune cells. The involvement of A_2A_ARs in tumor-associated lymphangiogenesis was uncovered: A_2A_AR-deficient mice exhibited diminished lymphangiogenesis in tumors and sentinel nodes, resulting in protection against metastasis. Lack of A_2A_ARs in both hematopoietic and nonhematopoietic cells contributes to this outcome [177].

Adenosine signaling has been reported to be linked with an unfavorable prognosis and might hold predictive value for the response to anticancer therapy. An analysis of the gene expression signature for adenosine signaling, primarily associated with A_2A_AR activation, establishes an adverse correlation between adenosine and overall survival, progression-free survival, as well as decreased efficacy of anti-PD1 therapy, among a cohort of patients treated with immune checkpoint inhibitors [178]. In patients with renal cell cancer (RCC), increased expression of A_2A_ARs in the primary tumors was associated with the presence of metastatic characteristics. Moreover, patients exhibiting lower A_2A_AR expression demonstrated improved response to therapy and prolonged overall survival [179].

Well-known and novel A_2A_AR antagonists have recently been tested in both preclinical settings and clinical trials. The novel A_2A_AR antagonist, DZD2269, demonstrated anti-tumor effects in syngeneic mouse models, particularly when used in conjunction with immune checkpoint inhibitors, radiotherapy, or chemotherapy. Preliminary results from a phase I clinical trial revealed that DZD2269 effectively suppresses pCREB within human T cells [180]. Similarly, other innovative A_2A_AR antagonists, AZD4635 and CPI-444 (Ciforadenant), exhibited the ability to diminish tumor growth while intensifying the effectiveness of checkpoint inhibitors in syngeneic tumor models [175,181]. Notably, mice that were rechallenged exhibited complete inhibition of tumor growth, highlighting the induction of systemic immune memory. Ciforadenant was subsequently tested in phase I clinical trial in a cohort of patients with advanced refractory RCC. The A_2A_AR antagonist demonstrated efficacy in immunotherapy-naïve patients and those resistant to anti-PD-L1 treatment, especially when combined with atezolizumab. Furthermore, an adenosine-related gene signature suggests the potential to identify patients who are likely to respond positively to adenosine pathway blockade-based treatments [182]. In castration-resistant prostate cancer (CRPC), colorectal carcinoma, non-small cell lung cancer (NSCLC), or other solid tumors, the safety and antitumor activity of AZD4635 were evaluated in a phase 1a/b open-label, multicenter study. AZD4635, whether used alone or together with durvalumab, exhibited favorable tolerability and showed no significant safety issues. Moreover, it demonstrated initial indications of clinical effectiveness in patients with metastatic CRPC [183]. Likewise, the A_2A_AR antagonist, Taminadenant, both with and without spartalizumab, was well tolerated in individuals with advanced NSCLC, where certain patients showed signs of clinical improvement.

Novel A_2A_AR antagonists for cancer immunotherapy continue to be synthesized and developed [184,185], including dual-acting compounds targeting both A_2A_ARs and other well-recognized cancer targets such as histone deacetylases [186,187] or CD73 [188]. In an attempt to mitigate the adverse effects of systemic A_2A_AR inhibition and facilitate tumor-specific delivery and activation of A_2A_AR antagonists, various innovative approaches utilizing nanotechnologies have been investigated. A photo-modulated nanoreactor was recently shown to induce oxygen-mediated reduction in A_2A_AR RNA expression within immune cells. By activating the nanoreactor exclusively within the TME using near-infrared radiation, this A_2A_AR inhibition led to substantial secretion of immune-related cytokines, enhancing anti-tumor immune responses and promoting tumor cell killing [189]. In a similar approach, on-site oxygen generation through hydrogen peroxide catalysis was harnessed to inhibit A_2A_AR responses. This method utilized macrophage membrane-coated mesoporous silica nanoparticles loaded with catalase, doxorubicin, and resiquimod. Oxygen-dependent A_2A_AR inhibition counteracted the immunosuppressive TME caused by hypoxia. This, in turn, prompted DCs to boost the immune response mediated by CD8^+^ T cells [190]. Furthermore, a polydopamine nanocarrier, concealed by an acid-sensitive PEG shell, was employed for delivering the A_2A_AR antagonist, SCH58261, to the tumor. Once it reaches the acidic TME, SCH58261 is selectively released within the tumor tissue, thereby enhancing the immune response against the tumor [191].

A prominent avenue in oncology’s advancing immunotherapy landscape involves chimeric antigen receptor (CAR) T cells [192]. Due to its immunosuppressive role, targeting A_2A_ARs has been confirmed as a successful approach for enhancing the effectiveness of CAR T cell therapy. The A_2A_AR antagonist reversed the decrease in CAR T cell proliferation and cytokine response triggered by agonists. However, it was not effective in restoring the cells’ cytotoxic functionality. The genetic removal of A_2A_ARs, achieved through either shRNA or CRISPR/Cas9, improves the in vivo effectiveness of CAR T cells, resulting in enhanced tumor eradication capabilities [193,194,195]. Recently, the dual A_2A_/A_2B_ AR antagonist, AB928 (etrumadenant), boosted CAR T cell cytokine production and proliferation, enabling the effective destruction of tumor cells in vitro and enhancing CAR T cell activation in vivo [196].

### 5.2. Recent Advancements in A_2B_AR Modulation for Cancer

A_2B_ARs are expressed in both immune and non-immune cells, and their activation has been associated with promoting cancer cell proliferation, tumor growth, the formation of metastases, tumor angiogenesis, and the suppression of immune system responses [197]. Recent studies further indicate that A_2B_ARs represent a potential target for cancer therapeutic intervention.

While the contribution of T cell A_2B_ARs to immunosuppression and tumor promotion was discovered to be minimal, the presence of A_2B_ARs on myeloid cells and antigen-presenting cells indirectly hindered CD8^+^ T cell responses and facilitated metastasis. The inhibition of A_2B_ARs through genetic or pharmacological means enhanced the effectiveness of adoptive T cell therapy [198]. Hypoxia is a prevalent characteristic of the TME, and there is substantial evidence indicating an increase in A_2B_AR expression upon the activation of HIF-1α. A recent study demonstrated the role of A_2B_ARs in hypoxia-induced breast cancer stem cell enrichment by activating PKC-δ/STAT3 pathway [199]. In addition to hypoxia, chemotherapy was also found to increase A_2B_AR expression, and its activation contributed to the expression of chemotherapy-induced pluripotency factors and the enrichment of breast cancer stem cells. As a result, in in vivo models of triple-negative breast cancer, genetic or pharmacological inhibition of A_2B_ARs resulted in a delay of tumor recurrence after discontinuation of chemotherapy [200]. Recently, highly effective A_2B_AR antagonists were synthesized and subjected to functional assessment in patient-derived tumor spheroid models. These novel compounds demonstrated the capability to restore T and NK cell proliferation, enhance the production of IFNγ and perforin, and promote increased infiltration of tumor-infiltrating lymphocytes [201].

Yu et al. demonstrated that A_2B_AR activation enhanced CD73 expression in cancer-associated fibroblasts, initiating a feedforward circuit to amplify the CD73-adenosine axis in the TME, which ultimately led to A_2A_AR-dependent inhibition of immune activation [202]. Elevated levels of CD73 were detected in activated CD8+ T cells in the pancreatic ductal adenocarcinoma TME. A recent study revealed that the A_2B_ARs on CD8^+^ T cells played a pivotal role in adenosine-mediated immunosuppression in pancreatic ductal adenocarcinoma models [203]. The CD73-dependent generation of adenosine through tumor-derived exosomes was demonstrated to drive the polarization of macrophages into an M2-like phenotype via A_2B_ARs, thereby facilitating the release of angiogenic factors. Consequently, targeting A_2B_ARs could potentially serve as a strategy to counteract tumor-derived exosome-induced tumor angiogenesis [204].

### 5.3. Recent Advancements in A_1_ and A_3_ AR Modulation for Cancer

While A_2A_ and A_2B_ ARs have long been regarded as the pivotal ARs responsible for orchestrating the immune response against tumors, emerging evidence is shedding light on the significant involvement of A_1_ARs as well.

In various immune-deficient cancer models, the deletion of A_1_ARs was shown to hinder tumor growth [205,206]. Nonetheless, recent findings have unveiled that the inhibition of A_1_ARs in immune-competent mice actually facilitated tumor immune evasion. The underlying mechanism for this phenomenon was identified as A_1_AR-mediated up-regulation of PD-L1 expression through the activation of ATF3. Notably, elevated A_1_AR expression was detected in tumor tissues of non-responder NSCLC patients when compared to those who responded to anti-PD-1 monoclonal antibody therapy [207]. A_1_AR was also identified as one of the most up-regulated genes in EGFR-mutant NSCLC associated with an immune-inert phenotype [208].

Different results were observed in HCC. Hypoxia-mediate adenosine generation mediated activated A_1_ARs, consequently fostering the accumulation of immunosuppressive plasmacytoid DCs. Employing an A_1_AR antagonist effectively impeded the migration of plasmacytoid DCs, leading to the suppression of tumor growth [209].

The overexpression of A_3_ARs is observed across nearly all types of cancer, suggesting their potential utility as a promising tumor biomarker. Furthermore, the activation of A_3_ARs could potentially introduce a novel approach to personalized cancer therapy [210]. Recently, elevated expression of A_3_ARs has been identified in tumor-infiltrating NK cells across various tumor types, significantly exceeding the expression levels found in peripheral NK cells [211]. The A_3_AR agonist, namodenoson (also known as CF102 or Cl-IB-MECA), exhibited encouraging outcomes in a phase I/II clinical trial involving advanced HCC and moderate hepatic dysfunction. The compound demonstrated favorable tolerance and displayed a modest enhancement in overall survival/progression-free survival, although it was not statistically significant [212]. Table 4 reports the latest in vivo studies on adenosine receptor modulation in cancer models.

## 6. Conclusions

Adenosine, its four receptor subtypes, and its metabolizing enzymes continue to be subjects of intensive research aimed at addressing numerous diseases. Over the past five years, significant progress has been achieved in understanding the mechanisms underlying adenosine-mediated modulation of crucial physiological functions and their roles in disease conditions. Given adenosine’s widespread involvement in the body, substantial advancements have been made in various fields where the modulation of ARs offers potential therapeutic avenues. These fields encompass CNS disorders, cardiovascular and metabolic conditions, inflammatory-based diseases, and cancer. However, the omnipresent nature of adenosine has posed challenges in the translation of AR-interacting drugs into clinical practice. As these challenges are gradually surmounted, the modulation of ARs could emerge as a transformative factor in treating diverse diseases. This notion is reinforced by the dedicated efforts to develop novel drug candidates centered around the manipulation of the adenosine system, as substantiated by a multitude of ongoing clinical trials (Table 5). As highlighted by this review, novel avenues have emerged for harnessing the full potential of ARs. Utilizing partial agonists, allosteric modulators, and biased agonists stands out as a promising approach to finely tune the modulation on these receptors. Moreover, the development of innovative drug delivery systems, coupled with the prospect of in situ activation, offers spatial and temporal control possibilities, thus contributing to the mitigation of adverse effects. Recent studies have also unveiled new insights into the intricate mechanisms regulated by ARs, paving the way for new therapeutic approaches across various diseases. Among these, the field of cancer treatment has witnessed remarkable activity, as evidenced by the elevated number of clinical trials in this area. Nevertheless, promising outcomes have surfaced in numerous other pathologies as well, bringing hope for the future application of therapies based on adenosinergic system modulation. 

## Figures and Tables

**Table 1:** The latest developments concerning adenosine receptor ligands in animal models of CNS pathologies.

Pharmacological Action	Compound	Experimental Model	Species	Effects
A_1_AR agonists	benzyloxy-cyclopentyladenosine	Neuropathic pain	rat	Analgesia [16]
	ENBA	Cerebral ischemia	rat	Microglia/macrophages proliferation reduction [66]
Dual A_1_/A_3_ AR agonists	AST-004	Cerebral ischemia	nonhuman primate	Ischemic damage reduction [71]
A_1_AR antagonists	DPCPX	Epilepsy	rat	Reversion of DBS impact on interictal epileptic discharges [78]
A_1_AR PAMs	MIPS521	Neuropathic pain	rat	Analgesia [14]
A_2A_AR agonists	ATL313	Neuropathic pain	rat	Anti-allodynia and anti-inflammation [18]
A_2A_AR antagonists	KW-6002 (istradefylline)	EAE model	mouse	Protection against T Cell Infiltration [58]
		Chemotherapy-induced cognitive impairment	mouse	Reversion of cisplatin-induced neurotoxicity [72]
		Retinal injury	mouse	Reduction of apoptosis of retinal ganglia cells [101]
			mouse	Regulation of retinal ganglion cell morphology [102]
	KW-6356	PD	marmoset	Motor disability reversion [45]
	SCH58261	SUDEP	mouse	Increase in theta and beta oscillations [79]
		Mania-like behavior	rat	Reduction of locomotor hyperactivity and risk-taking behavior [90]
	ZM241385	Craniocerebral trauma	mouse	Reduction of fear memory [62]
A_2A_AR PAMs	A_2A_RPAM-1	Mania-like behavior	mouse	Insomnia reduction [95]
A_2B_AR agonists	BAY60-6583	Focal ischemia	rat	Reduced brain damage [69]
A_3_AR agonists	Cl-IB-MECA	Glaucoma	rat	Preservation of retinal ganglion cell [99]
	IB-MECA	Chronic cerebral ischemia	mouse	Improved memory deficits [70]
	MRS5980	Traumatic brain injury	mouse	Reduction of tissue injury and cognitive impairment [63]
		Chemotherapy-induced cognitive impairment	mouse	Prevention of mitochondrial dysfunction and oxidative stress [73]
		Neuropathic pain	mouse	Anti-allodynia [23]
Non-selective antagonists	Caffeine	AD	mouse	Neuroprotection [41]

AD (Alzheimer’s disease), EAE (autoimmune encephalomyelitis), PAM (positive allosteric modulator), PD (Parkinson’s disease), SUDEP (sudden unexpected death in epilepsy).

**Table 2:** The latest developments concerning adenosine receptor ligands in animal models of cardiovascular and metabolic diseases.

Pharmacological Action	Compound	Experimental Model	Species	Effects
A_2A_AR agonists	ATL1223	Circulatory arrest and ECPR	pig	Reduction of systemic ischemia-reperfusion injury and ameliorated renal, hepatic, and cardiac injury [108]
A_2A_AR antagonists	SCH58261	High-sucrose diet	rat	Improved insulin response [120]
A_2B_AR agonists	BAY 60–6583	Obesity	mouse	Increased whole body energy expenditure [118]
A_2B_AR antagonists	MRS1754	High-sucrose diet	rat	Improved insulin response [120]

ECPR (extracorporeal cardiopulmonary resuscitation).

**Table 3:** The latest developments concerning adenosine receptor ligands in animal models of inflammatory and autoimmune pathologies.

Pharmacological Action	Compound	Experimental Model	Species	Effects
A_2A_AR agonists	CGS21680	Arthritis	mouse	Arthritis development inhibition [132]
		LES	mouse	Depletion of pathogenic lymphocytes and anti-nuclear antibodies reduction [135]
		Obesity-induced OA	mouse	Senescence markers reduction and improvement of mitochondrial stability and function [141,142]
				Increased autophagy and improved metabolic function in chondrocytes [143]
	PDRN	Acute lung injury	rat	Decrease in lung tissue damage, pro-inflammatory cytokines, apoptotic factors, and MAPK/NF-κB activation [152]
A_2A_AR antagonists	ZM241385	Sepsis	mouse	Enhanced survival by improving bacterial clearance [164]
A_3_AR agonists	CF101	OA	rat	Reduction of cartilage damage and pain [144]
	MRS5980	Lung fibrosis	mouse	Attenuation of lung stiffness and inflammatory/oxidative stress markers [161]
	MRS7344	Psoriasis	mouse	Hinder development of psoriatic-like characteristics [126]
A_3_AR/partial PPARγ agonist	LJ-529	Pulmonary Emphysema	mouse	Pulmonary function restoration, reduction of MMP activity and apoptosis [160]
Non-selective agonists	NECA	Airway inflammation	mouse	Reduction of innate lymphoid cells responses and inflammation [151]
Non-selective antagonists	Caffeine	Bronchopulmonary dysplasia	mouse	Decrease in lung injury, oxidative stress, and inflammatory infiltration [162]

LES (lupus erythematosus systemic), OA (osteoarthritis), PPARγ (peroxisome proliferator activated receptor γ).

**Table 4:** The latest developments concerning adenosine receptor ligands in animal models of cancer.

Pharmacological Action	Compound	Experimental Model	Species985	Effects
A_1_AR antagonists	DPCPX	HCC	mouse	Suppression of tumor growth [209]
A_2A_AR antagonists	AZD4635	Melanoma, colorectal carcinoma, fibrosarcoma	mouse	Decrease in tumor volume with enhanced T cell response [175]
	CPI-444 (ciforadenant)	Colorectal carcinoma, kidney tumor, melanoma	mouse	Diminution of tumor growth intensifying the effectiveness of checkpoint inhibitors [181]
	DZD2269	Melanoma, prostate cancer, and pancreatic cancer	mouse	Antitumor effects particularly when used together with immune checkpoint inhibitors, radiotherapy, or chemotherapy [180]
	SCH58261	CLL	mouse	Immune competence restoration by inhibiting the accumulation and differentiation of Treg cells [173]
		Breast cancer	mouse	Regression of primary tumor and inhibition of metastasis [191]
	ZM241385	Melanoma	mouse	Restoration of the functionality of tumor-infiltrating cytotoxic T cells [171]
Dual A_2A_/A_2B_ AR antagonists	AB928 (etrumadenant)	Colon carcinoma	mouse	Enhancement of CAR T cell activation [196]
A_2B_AR antagonists	Alloxazine	Breast cancer	mouse	Delay of tumor recurrence after discontinuation of chemotherapy [200]

CLL (chronic lymphocytic leukemia), HCC (hepatocellular carcinoma).

**Table 5:** Clinical trials involving adenosine receptor ligands that started in the last five years.

Pharmacological Action	Compound	Condition	Phase	NTC Number
A_1_ antagonists	PBF-680	Asthma	II	03774290
		COPD	IIa	05262218
A_2A_ agonists	Regadenoson	COVID-19	I/II	04606069
		Myocardial ischemia	I/II	04604782
		High grade gliomas	I	03971734
		Lung transplant	I	04521569
A_2A_ antagonists	Istradefylline	ALS	I/II	05377424
		PD	IV	05885360
		Cognitive impairment in PD	II	05333549
		Apathy in PD	Observational	05182151
	Ciforadenant (CPI-444)	Renal cell carcinoma	Ib/II	05501054
		Multiple myeloma	I	04280328
	TT-10	Solid cancers	I/II	04969315
	Inupadenant (EOS100850)	NSCLC	II	05403385
		Solid cancers	I	05117177
	AZD4635	Solid cancers	I	03980821
		NSCLC	I/II	03381274
		Prostate cancer	II	04089553
		CRPC	II	04495179
	DZD2269	CRPC	I	04634344
Dual A_2A_/A_2B_ antagonists	Etrumadenant (AB928)	Head and neck cancers	I	04892875
		Prostate cancer	II	05915442
		Liposarcoma	II	05886634
		Rectal cancer	II	05024097
		Urothelial carcinoma	II	05335941
		Gastrointestinal cancers	I	03720678
		Colorectal cancer	I/II	04660812
		CRPC	Ib/II	04381832
		CRPC	II	05177770
	M1069	Solid cancers	I	05198349
A_2B_ antagonists	PBF-1129	NSCLC	I	03274479
		NSCLC	I	05234307
	TT-702 (prodrug of TT-478)	Solid cancers	I/II	05272709
	TT-4	Solid cancers	I/II	04976660
A_3_ agonists	Piclidenoson (CF101, IB-MECA)	Ocular hypertension	I/II	04585100
		COVID-19	II	04333472
		Plaque psoriasis	III	03168256
	Namodenoson (CF102, Cl-IB-MECA)	NASH	II	04697810
		HCC/Cirrhosis	III	05201404
	FM-101	Ocular hypertension	I/II	04585100
		NASH	II	04710524
A_3_ antagonists	PBF-1650	Psoriasis	I	03798236
	PBF-677	Ulcerative colitis	II	03773952
Non-selective antagonists	Caffeine	Hypoxic-ischemic encephalopathy	I	03913221

Data from clinicaltrials.gov; ALS (amyotrophic lateral sclerosis), COPD (chronic obstructive pulmonary disease), CRPC (castrate resistant prostate cancer), HCC (hepatocellular carcinoma), NASH (nonalcoholic steatohepatitis), NSCLC (non-small cell lung cancer), PD (Parkinson’s disease).

## Data Availability

Not applicable.
